# Three-Dimensional High-Resolution Digital Inline Hologram Reconstruction with a Volumetric Deconvolution Method

**DOI:** 10.3390/s18092918

**Published:** 2018-09-03

**Authors:** Junseong Eom, Sangjun Moon

**Affiliations:** 1 Micro Mechanical System Technology Laboratory, Department of Mechanical Engineering, Korea Advanced Institute of Science and Technology, Daejeon 34141, Korea; ejs09@kaist.ac.kr; 2 Sensors and Aerosol Laboratory, Department of Mechanical, Aerospace and Nuclear Engineering, Ulsan National Institute of Science and Technology, Ulsan 44919, Korea

**Keywords:** digital holography, holography microscope, volumetric deconvolution, three-dimensional volumetric deconvolution

## Abstract

The digital in-line holographic microscope (DIHM) was developed for a 2D imaging technology and has recently been adapted to 3D imaging methods, providing new approaches to obtaining volumetric images with both a high resolution and wide field-of-view (FOV), which allows the physical limitations to be overcome. However, during the sectioning process of 3D image generation, the out-of-focus image of the object becomes a significant impediment to obtaining evident 3D features in the 2D sectioning plane of a thick biological sample. Based on phase retrieved high-resolution holographic imaging and a 3D deconvolution technique, we demonstrate that a high-resolution 3D volumetric image, which significantly reduces wave-front reconstruction and out-of-focus artifacts, can be achieved. The results show a 3D volumetric image that is more finely focused compared to a conventional 3D stacked image from 2D reconstructed images in relation to micron-size polystyrene beads, a whole blood smear, and a kidney tissue sample. We believe that this technology can be applicable for medical-grade images of smeared whole blood or an optically cleared tissue sample for mobile phytological microscopy and laser sectioning microscopy.

## 1. Introduction

The principle of in-line holographic microscopy was suggested by Gabor and is based on an electron holographic interference between a small object and illuminating wave, called the “electron interference microscope” [1]. Interference microscopy has been expanded into digital in-line holographic microscopy (DIHM) [2], supported by the development of digital detectors that have micron size pixels and a large image sensor, enabling the numerical calculation of diffraction and the reconstruction of a holographic image using various digital image processing techniques [3,4]. The merit of DIHM is its capability to be adapted to highly functional and simplified microscopes without complex physical optics and an expansive light modulator [5]. For example, the high-resolution digital image reconstruction approach for a wide field-of-view (FOV) image is one of the most influential methods in biomedical applications, such as the diagnosis and pathology for various lab chip platforms [6,7,8]. For the wide FOV of a whole slide digital image at a single shot, its resolution and size have an inverse relationship, which affects the total measurement time and cost of the imaging. The narrow FOV of the conventional microscopic imaging method to achieve the whole slide image requires a grid scanning and digital stitching method, with a high magnification factor imaging resolution, to expand the FOV. The digitalized image and its optical calculation of the analytical wave equation, Fourier optics, have broken through the limitations of the conventional microscope, such as resolution enhancement beyond the diffraction limit [9], high-resolution [10], wide field-of-view imaging from a 2D planar hologram [11], on-chip differential interference contrast microscopy [12], and wide field-of-view lens-free fluorescent imaging [13].

Recently, the DIHM has been introduced into 3D imaging applications, providing new approaches to obtaining a volumetric image with both a high resolution and large FOV size, which allows the physical limitations and focal depth to be overcome. Based on the 2D DIHM technology and its various merits, 3D section imaging technologies relating to volumetric samples have been suggested for a thick clear tissue, widespread whole blood cells, and polystyrene beads on a sliding glass, i.e., 3D imaging of CLARITY tissue [14], three-dimensional profiling and tracking [15,16], angled tomography [17], 3D image distortion compensation [18], and wide-field pathology slide imaging [19]. There are two significant challenges that must be overcome to achieve a wide FOV high resolution 3D image: Phase recovery and spatial under-sampling, based on an iterative reconstruction with a single shot hologram [20,21], multi-angle [22], multi-height [23], spatial shift [24], colorization [25] for color artifacts [26], a scattering medium [27], a diffuser [28], a scattering mask [29], and multi-wavelength [30]. In this 2D image reconstruction and phase recovery, twin image and spatial aliasing signals, along with other digital artifacts, were solved using each separate technology or an integrated propagating phaser approach based on this idea [31]. These approaches show a significant improvement in resolution and a significant elimination of reconstruction artifacts, which can aid the numerical compensation of a sub-pixel super-resolution and three-dimensional volumetric image.

However, during the sectioning process of the 3D image generation, the out-of-focus reconstructed wavefront becomes a significant impediment to obtaining clear 3D cells in the sectioning of the thick biological sample [14] or particle images [15] and in its application for flow visualization [32]. Except for the off-axis interferometer approach [33], there are three approaches to solving the problem. One of these strategies uses a 2D hologram, which can be calculated from the iterative phase matching method. This wavefront reconstruction approach can generally be adopted in super-resolution image reconstruction. Another simple way to find a focused depth is to use image processing with sharp contrast detection to determine a Z-depth profile in the noisy out-of-focus image. The other approach is 3D deconvolution method, which is to remove artifacts from the out-of-focus image at the target Z-depth focusing plane [34,35]. The 3D deconvolution method can reduce the artifacts from the out-of-focus signal using the low-resolution reconstructed wave-front, although it is necessary to improve the noise suppression and resolution enhancement in the case of a super-resolved holographic image. Since 3D deconvolution methods target a simple particle sample, i.e., a bead and low-resolution holographic image, as described in previous reports [34,35], the 3D deconvolution method cannot reflect the real situation of a medical sample and application, such as whole blood and pathology.

**Figure 1:**
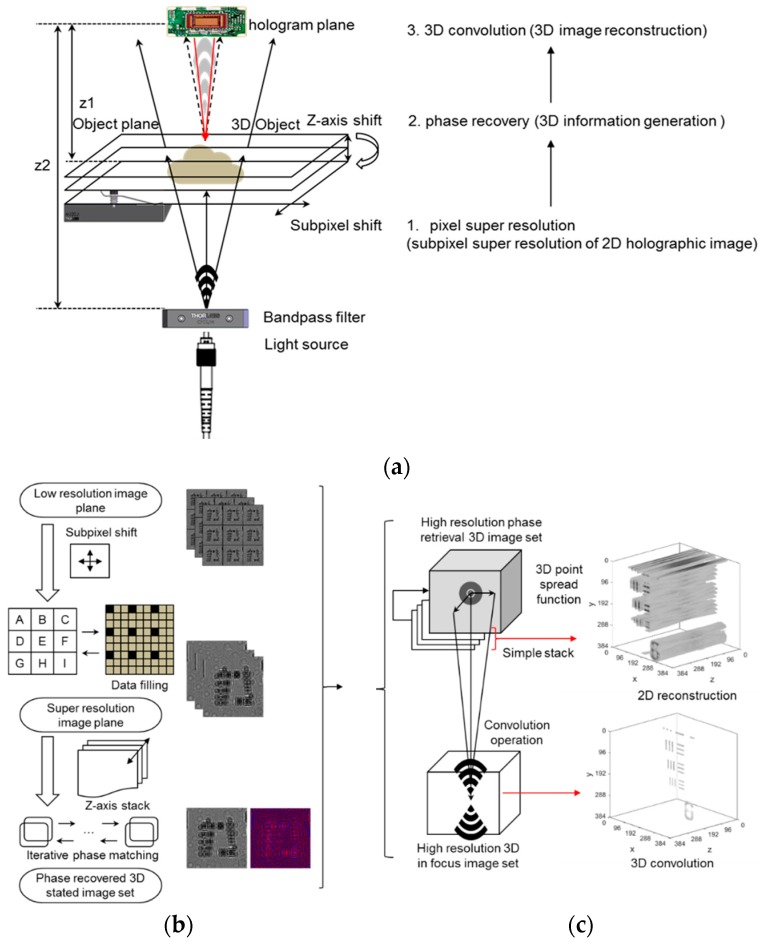
A schematic description of the experiment setup and the proposed algorithm for three-dimensional super-resolution digital in-line holographic microscopy using a volumetric deconvolution approach. (**a**) A low-resolution image acquisition apparatus using the in-line holographic approach. By using a narrow bandpass filter and light source, connected with an optical fiber, temporally coherent light is propagated along an object plane and generates an interference image at an image sensor, i.e., detector plane. The object plane moves with the sub-pixel shift in the *X* and *Y* direction for the pixel-super-resolution process and *Z*-axis shift for multi-height phase recovery. (**b**) Phase recovered high-resolution hologram image generation. For high-resolution three-dimensional deconvolution, a phase recovered complex hologram image is generated by the pixel-super-resolution process and multi-height phase recovery algorithm. (**c**) Three-dimensional volumetric imaging, which is constructed by the deconvolution process. The complex hologram image is back propagated to a series of *Z*-axis distances to generate a *Z*-stacked complex object field. Then, a clearly focused high-resolution volumetric object image is constructed by the three-dimensional deconvolution process with a simulated complex point spread function.

Here, we present a method to improve the 3D object image quality by the volumetric deconvolution and phase recovery of a super-resolution 2D holographic image. This approach consists of two steps to obtaining fully resolved 3D volumetric images. The three-dimensional image reconstruction, from low resolution to high resolution, is based on the conventional phase retrieval super-resolution and 3D volumetric deconvolution approach to rebuilding a real object from the image plane, as shown in Figure 1. First, the super-resolution image is obtained using low-resolution subpixel movements and a phase retrieval algorithm, as shown in Figure 1b. Second, the volumetric object is reconstructed using the 3D volumetric convolution of the super-resolution hologram image, which acts as a spatial filter, as shown in Figure 1c. Based on this approach, we demonstrate that a high-resolution 3D volumetric image, which significantly reduces wavefront reconstruction and out-of-focus artifacts, can be achieved. We believe that it can produce medical-grade images of smeared whole blood or an optically cleared tissue sample for mobile phytological microscopy or laser sectioning microscopy.

## 2. Materials and Methods

### 2.1. Optical Imaging Setup

**Figure 2:**
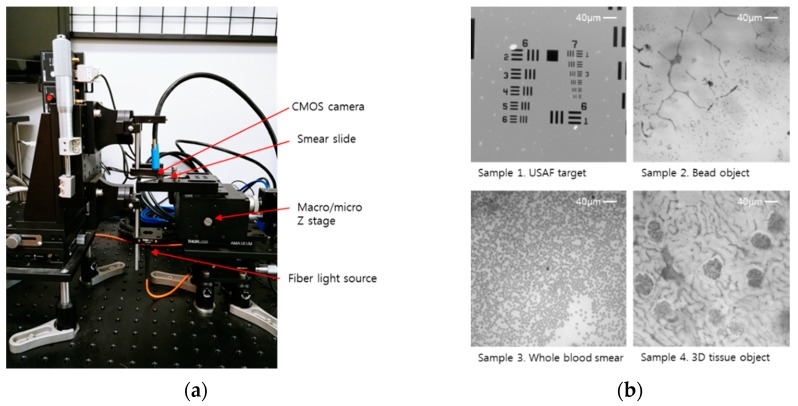
Image acquisition apparatus for the inline holographic microscopy and four different test samples. (**a**) From the fiber light source, a point, like a source, emits on the bottom of the sliding glass as a reference light. Between the slide and cover glass, a thin layer of the sample object scatters the reference light, which creates an interference pattern. The interference pattern, e.g., the hologram image, is propagated on a CMOS sensor surface. The CMOS camera (10.7 Mp, 1.67 µm pixel size) is placed on the top of the cover glass to minimize the point scatter path and records the hologram image. To obtain the subpixel resolution image, the slide holder translates the submicron range by the nanopositioning XYZ stage. (**b**) Four different test samples are used to validate the 3D image reconstruction, i.e., the USAF target for resolution validation after the reconstruction process, a micrometer-size bead, smeared whole blood, and a kidney tissue sample. A conventional microscope takes the 10X magnified images. The scale bar is 40 µm.

The optical imaging setup to capture the hologram of a sample is shown in Figure 2a. A fiber-coupled LED, with a peak wavelength of 530 nm (M530F2, Thorlabs, Inc., Newton, NJ, USA), is used as a partially coherent light source. At the end of the fiber, the light is spread as if over a pinhole, with a 50 μm diameter and a 0.22 numerical aperture. To enhance its temporal coherence, a narrow band filter, which has a center wavelength of 532 nm and a 10 nm full-width bandwidth (FL532-10, Thorlabs, Inc.), is placed in front of the light source. A monochrome CMOS imaging device (DMM 27UJ003-ML, The Imaging Source, Bremen, Germany), which is held by a microscope slide holder (MAX3SLH, Thorlabs, Inc.), is used to acquire holographic images of the sample. It is attached to a motorized stage with piezo actuators (MAX341, Thorlabs, Inc.) for slide scan and sub-pixel movement. All devices are aligned to obtain an accurate Gabor hologram of the sample. The distance between the light source and the sample is great enough (Z2 = 80 mm in Figure 2a) to make the wave-front of the light appear as a plane wave just before it is illuminated in the sample. On the other hand, the sample and the imaging device are placed as close as possible, which has two benefits. One is a decrease in the numerical error of reconstruction, and the other is the maintenance of the magnification (F = Z1/Z2) of the hologram image at almost one unit. With this property, the FOV of the hologram image is about 29.85 mm^2^, while the imaging sensor has a pixel size of 1.67 µm and 10.7 megapixels (3872 × 2764 pixels).

### 2.2. Sample Preparation

Four samples, which are used to demonstrate the efficiency of the resolution enhancement, phase recovery, and 3D deconvolution algorithm for digital in-line holographic imaging, are shown in Figure 2b. Four different samples are used to validate the 3D image reconstruction, i.e., the USAF target for resolution validation after the reconstruction process, a micrometer size bead, smeared whole blood, and a tissue sample for a different refractive index matching. The USAF-1951 target is the first sample that, this paper suggests, demonstrates the whole process and shows the optical resolution of its result. Micro-beads with a size of 1 µm are sparsely placed on both sides of a glass slide, which is used as a sample for particles of two different focal planes. A commercial human blood smear, Wright’s stained slide sample (#31-3158, Carolina Biological Supply Company, Burlington, NC, USA) and kidney tissue (#31-5788, Mammal Kidney 7 µm H&E, Carolina Biological Supply Company) are representative biological samples of small-size features.

### 2.3. Hardware and Software for Computation

All computation in this paper is done on a personal computer with the following specifications: Intel Core i7-7700HQ CPU @ 2.8GHz, 16GB DDR4 RAM, and Nvidia GTX1070 GPU. The software is written in MATLAB (ver. 2017a), utilizing CUDA acceleration for pixel-super-resolution and fast Fourier transform calculation. The total time consumption for each procedure, with the 6 × 6 grid and six heights of low-resolution hologram images (256 × 256 pixels, 216 images), are as follows: 15.6 s for pixel-super-resolution, 9.4 s for multi-height phase recovery, and 17.2 s for three-dimensional deconvolution.

### 2.4. Pixel-Super-Resolution (PSR)

Due to the limited pixel size of the imaging device, there exists a theoretical sampling resolution limit, which is the same as the size of a pixel, i.e., 1.67 µm. To overcome this limitation, various methods are introduced in relation to image processing. One promising approach is suggested for digital in-line hologram microscopy, i.e., the pixel-super-resolution process. Multiple low-resolution images are acquired with a subpixel shift in both the *X* and *Y* direction, moving the sample in a rectangular grid whose size is equal to the pixel size divided by the up-sampling factor. Since the factor is 6, the grid size is 0.278 µm, and 36 images are captured at one *Z*-axis position. These images are synthesized into one high-resolution image, with a fast-computational algorithm that is proposed in previous works of the Ozcan group [36].

### 2.5. 3D Phase Recovery and Lens-Free Image Reconstruction

The high-resolution hologram image is digitally back-propagated to reconstruct the focused sample image using the angular spectrum method, which is introduced by Latychevskaia et al. [4,21], but the reconstructed sample image contains a twin image diffraction pattern, which is caused by the loss of phase data in hologram images. To remove the reconstruction problem, hologram images are captured at several *Z*-axis positions for the multi-height phase recovery process, which is introduced by Ozcan et al. [37]. The phase data can be solved by multiple intensity images at different sample-to-detector distances or heights. At each height, hologram images are captured with the rectangular grid for PSR, and this is repeated at six different heights. Those six PSR hologram images are used for the iterative process of phase recovery. The first image, which is the closest to the imaging device, is chosen for the initial step, with zero phase. The image is digitally propagated at the next height, where its calculated amplitude is averaged with the captured actual image amplitude, which is the square root of intensity, and the phase remains unchanged. The difference between two heights is selected to be 20 µm, and the motorized sample stage is programmed to achieve this. The exact value of the height, however, is determined by the autofocusing algorithm, which maximizes the image sharpness factor, with a gold section search algorithm. The hologram image is propagated, and the average amplitude at each height, sequentially to the last image in the same work, in the opposite direction towards the first image, is calculated. These works consist of one iterative process of the phase recovery and can be repeated until the normalized difference between each sequential step is below a small value. The last resultant image and its phase data can be propagated and autofocused to obtain a final sample image, which has no, or less, twin image artifact. 

### 2.6. 3D Deconvolution Method

To achieve 3-dimensional scattering of the object function of the sample, 3D deconvolution is performed, with the reconstructed field from the hologram image and a point spread function, as suggested by Latychevskaia et al. [34,35]. The reconstructed object field is a simple stack of backward reconstructed images with a given *Z*-directional step size. This volumetric image is a measured datum of a 3-dimensional object, acquired at several focal planes. As the object $O\left(\vec{r}\right)$ can be represented as a sum of point scatterers $\delta \left(\vec{r}\right)$ in Equation (1), the measured image $M\left(\vec{r}\right)$ is written as a convolution of the object and the point spread function $\mathrm{PSF}\left(\vec{r}\right)$, as in Equation (2). In this section, an effective method to achieve the unknown object function $O\left(\vec{r}\right)$, i.e., 3-dimensional deconvolution, is introduced:

(1) $$O\left(\vec{r}\right)=\int O\left(\vec{s}\right)\delta \left(\vec{r}-\vec{s}\right)d\vec{s}$$

(2) $$M\left(\vec{r}\right)=\int O\left(\vec{s}\right)\mathrm{PSF}\left(\vec{r}-\vec{s}\right)d\vec{s}=O\left(\vec{r}\right)⊗\mathrm{PSF}\left(\vec{r}\right)$$

The point spread function can be calculated from a point source or a point-size blocker by the forward and backward propagation of a complex wave, using the angular spectrum method. For the following deconvolution calculation, the point spread function is simulated for the same three-dimensional volume of the measured object image.

From the convolution theorem, the given Equation (3) can be solved by direct division, as in Equation (4). The measured data, however, always contain a small amount of noise, which can be estimated as white noise with the Gaussian distribution and should be controlled by a small constant parameter β. The convolution approach is a linear restoration method and has some limitations, such as neglecting frequencies from the optical transfer function and insufficient constraints concerning physically impossible data. To improve the result of deconvolution without these disadvantages, the Gold’s method, which is a nonlinear iterative algorithm, is introduced:

(3) $$FT\left[M\left(\vec{r}\right)\right]=FT\left[O\left(\vec{r}\right)]\cdot FT[\mathrm{PSF}\left(\vec{r}\right)\right]$$

(4) $$O\left(\vec{r}\right)=FT^{-1}\left[\frac{FT\left[M\left(\vec{r}\right)\right]}{FT\left[\mathrm{PSF}\left(\vec{r}\right)\right]}\right]\approx FT^{-1}\left[\frac{FT\left[M\left(\vec{r}\right)\right]\cdot {\left(FT\left[\mathrm{PSF}\left(\vec{r}\right)\right]\right)}^{*}}{{\left|FT\left[\mathrm{PSF}\left(\vec{r}\right)\right]\right|}^{2}+\beta }\right]$$

The iterative method for 3D deconvolution estimates the real object, using PSF to simulate the measured data through the optical system, which can be compared with the real measured data. An error between the simulated data and the observed data is used to generate a more accurate estimation of the object in the next step of the iteration. The algorithm is as follows. The first guess of the object $O^{\left(0\right)}\left(\vec{r}\right)$ is given as the measured data, i.e., Equation (5). The measured image is blurred in the *Z*-direction by the convolution of the point spread function, i.e., Equation (6). The result is compared to the observed image data, and the object data in the next step is calculated. This sequential procedure is repeated until the object data produce suitable results, i.e., Equation (7). Applying too many iteration steps results in an over-fitted object, since the method is a statistically most likelihood algorithm:

(5) $$O^{\left(0\right)}\left(\vec{r}\right)=M\left(\vec{r}\right)$$

(6) $$M^{\left(k\right)}\left(\vec{r}\right)=O^{\left(k-1\right)}\left(\vec{r}\right)⊗\mathrm{PSF}\left(\vec{r}\right)$$

(7) $$O^{\left(k\right)}\left(\vec{r}\right)=O^{\left(k-1\right)}\left(\vec{r}\right)\cdot \frac{M\left(\vec{r}\right)}{M^{\left(k\right)}\left(\vec{r}\right)}\approx O^{\left(k-1\right)}\left(\vec{r}\right)\cdot \frac{M\left(\vec{r}\right)\cdot {\left(M^{\left(k\right)}\left(\vec{r}\right)\right)}^{*}}{{\left|M^{\left(k\right)}\left(\vec{r}\right)\right|}^{2}+\beta }$$

## 3. Results and Discussion

### 3.1. Subpixel Super-Resolution and Auto-Focused Z-Stack Reconstruction

**Figure 3:**
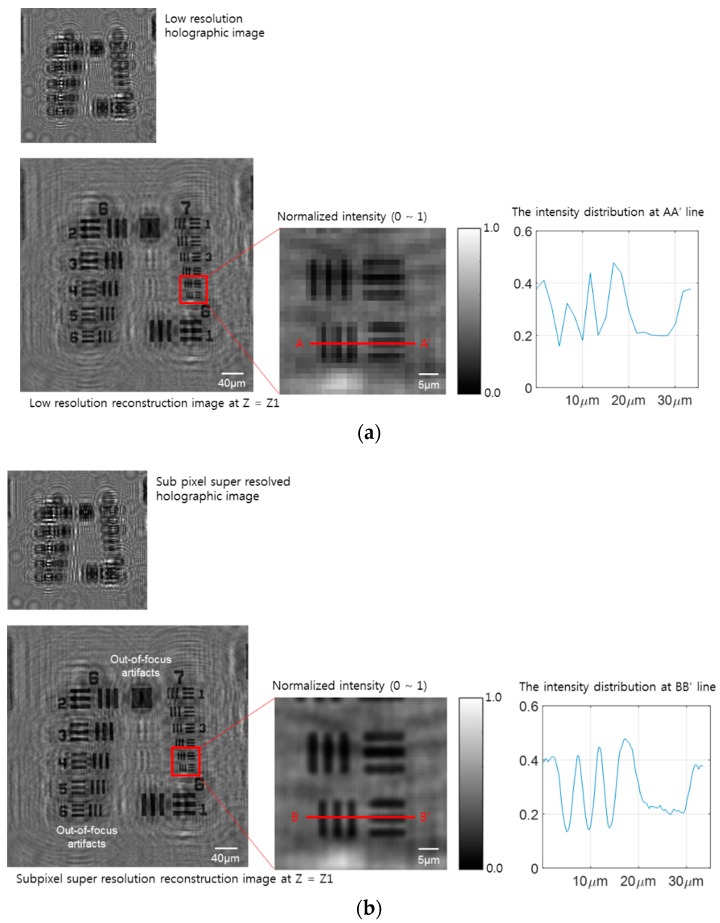
Two image reconstruction results of the USAF object from a set of low-resolution hologram images. (**a**) From the low-resolution hologram in the left upper inset figure, an object image is regenerated with a focused distance by an image processing algorithm, i.e., the angular spectrum method based on Fourier optics. Intensity distribution following the *X*-axis cutting line is normalized to its maximum value. The minimum resolution is a 2 µm FWHM in the USAF target group 1. (**b**) A sub-pixel super-resolution image from the low-resolution set of 36 images. The reconstructed image shows out-of-focus artifacts and a twin image artifact. However, the resolution enhancement is five times greater without the phase recovery step. The line, AA’ and BB’, graph shows a higher contrast, compared with the low-resolution case, and a 6X ideal resolution enhancement.

Resolving the power of the digital in-line holographic microscope (DIHM) using subpixel super-resolution is evaluated with the USAF-1951 resolution target for the reference scale of a purely absorbed sample. Because the pixel size of the monochrome imaging device is 1.67 µm, and the magnification factor of the microscope follows the principle of in-line holographic imaging (its setup is shown in Figure 1), the theoretical diffraction limit of the low-resolution image is 1.67 µm. The resultant power of the low-resolution hologram image and the intensity profile of a magnified region is represented in Figure 3a at group one of the USAF-1951 target. The focal distance of the target is calculated with an autofocusing algorithm, using a Sobel filter to find a sharp-edge focused plane [19]. The magnified image shows the minimum detection length, a 3 µm full-width half maxima (FWHM), as shown in the magnified figure and intensity distribution graph through an AA’ sectioning line, related to its pixel and magnification factor. For the pixel-super-resolution process, a set of 36 low-resolution images, with sub-pixel movements through a local sample coordination *X*- and *Y*-direction, are used to generate a high-resolution image with a pixel size of 0.278 µm. The theoretical resultant power is multiplied six times, as shown in Figure 3b. By applying the subpixel algorithm, the reconstruction artifacts can be reduced and compared to the low-resolution image reconstruction, but there are still reconstructed and out-of-focus artifacts at the focal plane, which are generated by some dust on the target surface, creating unwanted background interference pattern.

### 3.2. Phase Recovered Hologram Image for Both a Single-Shot and Z-Stacked Image Set

Not only for the 2D hologram resolution enhancement, but also for the 3D image reconstruction, a set of multi *Z*-axis holographic images are taken through the six-movement of the *Z*-axis, from 0 mm to 0.6 mm, with a 0.1 mm step depth, following the materials and method section. To achieve a qualified phase recovered hologram image, the *Z*-axis shift of the sample plane is recommended to be at least three steps in the *Z*-axis distance and separated by about 10 to 20 µm. Six images are used for this work, and more *Z*-axis shift steps lead to a more accurate result, but more time is required for an accurate calculation. If the distance between the sequence of the *Z*-axis step is too small, i.e., sub-micron or about 1 micron, the small intensity transition between two different distances makes the phase recovery process unreliable.

**Figure 4:**
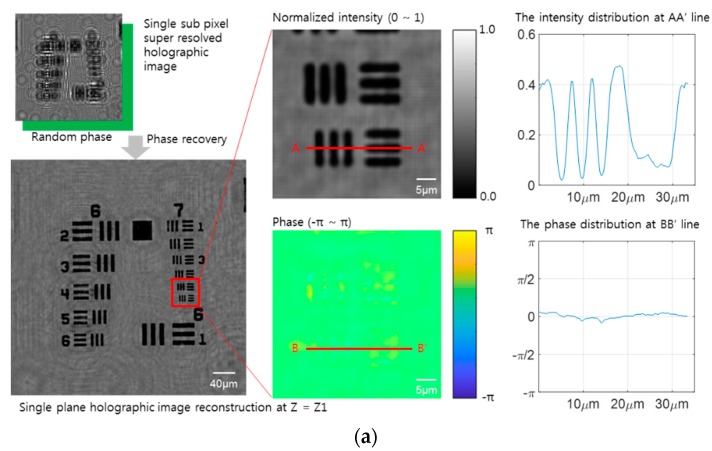

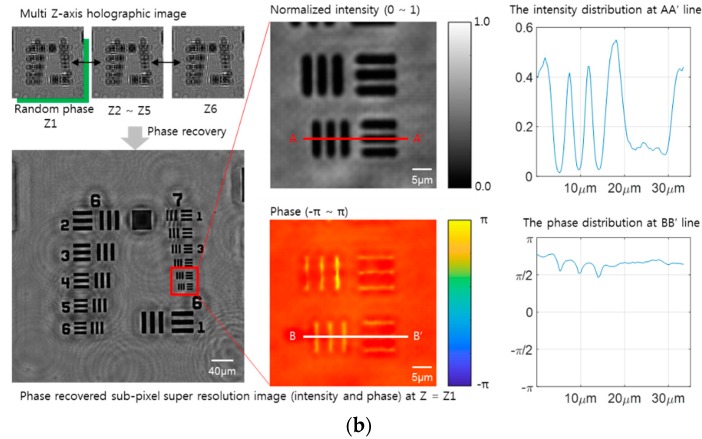
Comparison of two phase recovery algorithms, a single-height and an iterative multi-height phase recovery process. (**a**) A single height subpixel super-resolution hologram image and its reconstruction. The magnified image shows the intensity and phase after single-step phase recovery, which is set with a random phase value. The twin image artifacts are reduced in the reconstructed image and compared with no phase recovery. (**b**) An iterative phase recovery image using a set of multi-height holographic images. With a random phase image from the first *Z*-height hologram, the forward propagation performs and compares the next *Z*-height hologram intensity to renew the phase using the iterative reconstruction method. The phase recovered super-resolution hologram shows a significant reduction of the twin image artifacts. The full information of the hologram intensity and phase shows a similar profile following the sliced graphs, which are utilized for the 3D image generation. The effect of removing artifacts through the phase recovery process shows a significant enhancement in resolution.

The super-resolution images are used to determine the phase, which can aid the refinement of the 3D image and reduce the reconstruction artifacts. One of the simple methods to determine 3D phase information from the 2D intensity of the hologram is an iterative phase reconstruction approach with an edge mask, using a Gaussian blurred kernel, which is introduced by Tatiana Latychevskaia et al. [21]. The other approach is based on the *Z*-axis stacked holographic image, which has real partial information along the *Z*-depth. The different *Z*-depth hologram is used as reference intensity and constraint information during the iterative phase reconstruction from arbitrarily chosen initial phase information. The intensity and phase profiles, along the *X*-axis over the target pattern with the smallest width, of these two approaches are compared, as shown in Figure 4a,b. These results show the enhancement of the resolution power and artifact elimination in an auto-focused plane. Both the subpixel super-resolution hologram and *Z*-stacked phase retrieval approach reduce reconstruction artifacts and enhance the resolution by up to two times. However, one of the promising results, concerning the resolution enhancement and phase recovery effects of these approaches, is reported by the Zuo group [23], who reported that only a *Z*-stacked image set was needed to achieve both super subpixel resolution and phase recovery. The approach makes the imaging apparatus a simple and a mobile platform compared with other 3D image generation methods, such as multi-angle [22] and multi-wavelength [30] holographic image generation to minimize the movable parts of an apparatus. However, only the phase recovery cannot eliminate the out-of-focus artifacts that are generated from the different *Z*-depth objects; the USAF target does not have a different focal object acting as a 2D planar object.

### 3.3. 3D Sectioning Using the Conventional Auto-Focus Algorithm and 3D Deconvolution Approach

**Figure 5:**
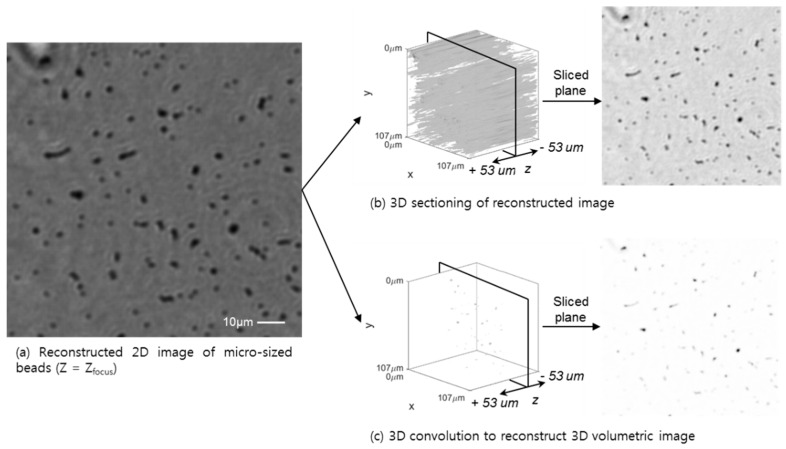
Planar and volumetric representation of the deconvolution results of the micro-sized beads sample (1 um diameter) are presented, both for the 2D reconstruction, i.e., slide sectioning, and 3D convolution, with a high resolution. (**a**) The planar image at the focused plane of the object (Z_focus_ = 721 um). (**b**) The stack of each focused plane generates a 3D volume representation. The high-resolution 3D object image has a pixel-super-resolution that is six times higher, processed from 36 low-resolution images. The 2D and stacked 3D image shows background artifacts, which reduce the 2D and 3D image contrast. (**c**) Volumetric deconvolution result of the object. Intensity distribution along the Z-direction through the full thickness shows a clear 3D image with reduced artifacts and increased 3D image contrast.

The 3D deconvolution method is applied to the acquired high resolution and phase recovered hologram image of the micro-size beads on a slide, which results in an effect of a volumetric object, as shown in Figure 5. The hologram images to reconstruct the measured data $M\left(\vec{r}\right)$ are prepared for the phase, recovered with six times pixel-super-resolution hologram images at six different heights, evaluated from 216 (36 × 6) low-resolution images for the two cases. The high-resolution phase recovered holographic image set is used for both a tomographic and a 3D volumetric reconstruction method. First, the 3D sectioning image is generated with an angular spectrum based back propagation method and is stacked with the different heights of the object, as shown in Figure 5b. Second, the volumetric object function is evaluated by the iterative method using Equation (7), with β = 0.001. After step (i), at every loop of iteration, the absolute value of the intermediate function $M^{\left(k\right)}\left(\vec{r}\right)$ is normalized, and its maximum and minimum are matched with the absolute value of the measured data $M\left(\vec{r}\right)$. Since the pixel size of the high-resolution image is 0.278 µm, the 1 µm-size beads show as four pixels-size points in each image, as shown in Figure 5c. In both cases of the tomographic reconstruction and 3D volumetric reconstruction, a planar image at the selected focused plane and a volumetric plot through the entire *Z*-depth are evaluated to compare the artifact elimination efficiency of the out-of-focus image. The intensity profiles along the *Z*-direction, around the focused plane and the normalized background intensity, indicate that the 3D volumetric deconvolution is more suitable for the 3D-shape sample, as shown in Figure 5c (inset graph and image). The 3D volumetric deconvolution image reveals a clear phase change and outline of the sample, not only through the 2D spatial plane, but also through the entire Z-depth. Unlike the tomographic image reconstruction by simple point scattering through the angular spectrum backpropagation approach, the 3D volumetric deconvolution shows a pinhole-like wavefront confinement function, which is realized by simple 3D deconvolution, with an arbitrarily chosen point spread function. Despite reducing the out-of-focus artifacts of the purely absorbable or reflective object, the suggested algorithm should be adopted for uneven phase-shifting materials, such as a whole blood cell and a thick tissue sample. The uneven phase-shifting and thick biological samples are reported by the Ozcan group [14] based on a conventional tomographic sectioning approach, which shows the high level of background noise stemming from the thick layer of out-of-focus planes. 

### 3.4. 3D Deconvolution Approach for a Whole Blood Smear and Biological Tissue Sample

**Figure 6:**
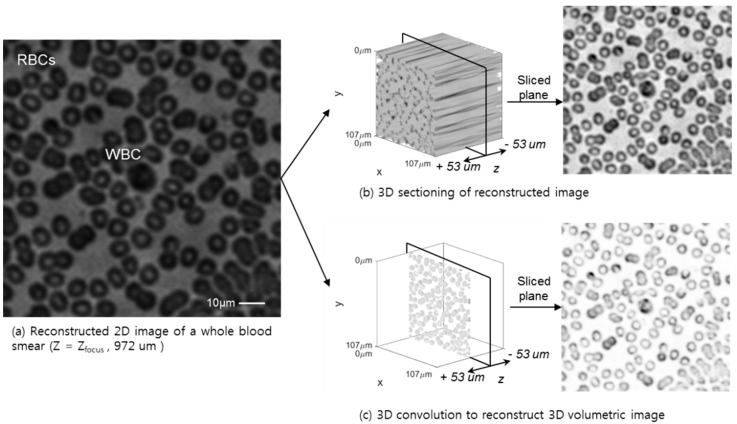
For real biological sample tests, whole blood images at Z_focus_ = 972 um (**a**), are reconstructed by 2D (**b**) and 3D (**c**) volumetric deconvolution. From a 2D hologram image, high-resolution 3D whole blood cells can be identified as red blood cells and white blood cells, which show a clear image of a nucleus. Complete information about the intensity and phase in the cell structure also aids identification according to the refractive index. The 3D convolution method shows more of the volumetric feature of the blood cells than the 2D approach.

**Figure 7:**
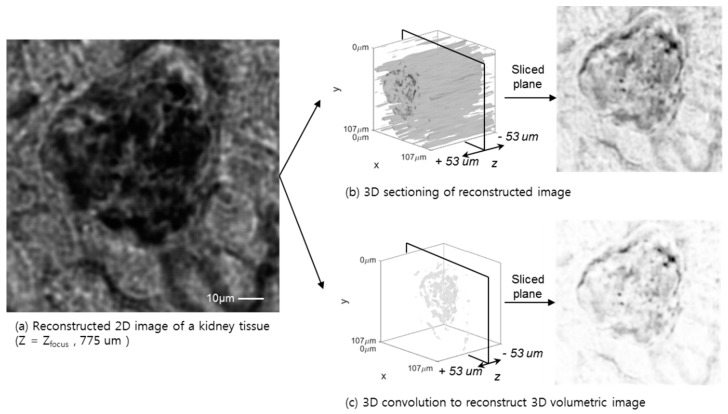
A kidney tissue image at Z_focus_ = 775 µm (**a**), is reconstructed by 2D (**b**) and 3D (**c**) volumetric deconvolution which is similar to the whole blood smear image. The kidney tissue image shows the morphology of the kidney cells and its identical structure after the 3D volumetric deconvolution.

The 3D volumetric reconstruction results for a whole blood smear, using a conventional tomographic 3D sectioning based on the angular spectrum backpropagation and auto-focusing algorithm, are shown in Figure 6. These two approaches for whole blood smear samples show a clear difference in the cell identification process, including a white blood cell context, like nuclei, and donut-shape red blood cells. Even in the case of whole blood, which has a high density of cells, each cell can be identified and counted, not only through the 2D plane, but also with a 3D depth profile. To apply the algorithm to a thick phase-shifting sample, a kidney tissue sample is reconstructed, as shown in Figure 7. The tissue fills the entire field-of-view of the sensor surface, which acts as a high level random phase shifter without a purely transitive slide glass background. The entire sample has a discontinuous phase changer, although the 3D volumetric reconstruction shows a transparent background through the entire 2D plane and *Z*-depth, which also eliminated the reconstruction and out-of-focus artifacts. 

By stacking and regenerating the 3D image, a bright and sharp-edge 3D image can be obtained. This approach might be more useful in fluorescent 3D imaging of a wide field-of-view application, since the fluorophore acts like a point light source that generates a higher level of out-of-focus scattering light in the focused plane.

## 4. Conclusions

The suggested 3D convolution method is widely adopted for confocal microscopic image reconstruction, which can find a focal depth definition by aiding the optical hardware, pinhole or pinhole array. However, for the mobile and simplified microscope apparatus in the medical field, the confocal microscope approach has lots of limitations in relation to achieving high-resolution 3D volumetric biological sample images, such as the price, volume, and operation of the microscope instrument. The DIHM demands high-performance computational power to calculate Fourier optics, although it eliminates complex optical components and the skillful operation of the microscope to achieve a repeatable high definition 3D image along the entire sample area, i.e., slide glass scanning. Following the suggested approach of the 3D convolution method, a mobile platform with wholly automated sample handling and 3D volumetric reconstruction from the focused plane, without further manipulation of the sample, can be achieved.

The Zuo group introduced *Z*-stacked hologram images to obtain high-resolution holographic images, with sub-pixel super-resolution and phase recovery, without a sub-pixel shift [23]. This approach can be adapted for simplifying the apparatus without using the nanometer resolution of the *XY* stage to obtain a sub-pixel hologram image. This result shows that the phase recovery procedure can be achieved with a sub-pixel resolution and the random motion of the sample during *Z*-stage motion. Moreover, the other technique is introduced to simplify the in-line holographic microscope with LED-based light source movement, wavelength scan, and multiple color imaging. Based on these technologies, the 3D deconvolution method will face a challenge in the reconstruction of the 3D volumetric image, not only in minimizing 2D high-resolution artifacts, but also in minimizing 3D out-of-focus image artifacts.

The suggested algorithm is based on a two-step subpixel super-resolution image reconstruction and 3D convolution procedure to achieve a high-resolution 3D image. To save the computation resources and time, the separated two-step calculation might be integrated into a one-step 3D image reconstruction method, following previous work, introduced to integrate the artifact removing algorithm, by the Ozan group [31]. For the one-step 3D image reconstruction, the auto-focusing step, which is used in the phase retrieval algorithm, can be eliminated, and the post process is to find a focus plane from the 3D reconstructed volumetric image data by minimizing out-of-focus image artifacts. This approach can aid the development of a mobile type of pathology microscope based on reduced computational power, which can be adopted with a statistical determination of phase or multiple low-resolution holographic images for deep learning [38]. By adopting 3D convolution and Fourier optics to reconstruct the original sample object, a scanning microscope for a mobile platform can be developed in the medical diagnosis field by reducing complex optical parts. In future, we will try to obtain the 3D image using the mobile platform from the medical pathology slide using high-resolution 3D imaging.
